# Atrial fibrillation phenotypes derived from multimodal data exhibit distinct electrophysiological substrate characteristics, symptom trajectories, and ablation outcomes

**DOI:** 10.1093/ehjopen/oeag162

**Published:** 2026-09-28

**Authors:** Neil Bodagh, Vinush Vigneswaran, Ali Gharaviri, Irum Kotadia, Magda Klis, Nilanka N Mannakkara, Kestutis Maciunas, Arno Von Kietzell, Megan Bell, Hayley Cuthbert, Matthew Wright, John Whitaker, Christopher A Rinaldi, Anoop Shetty, Shaumik Adhya, Gurpreet Dhillon, Roy Jogiya, Una Buckley, David Jones, Zhong Chen, Amedeo Chiribiri, Neil R Grubb, Chris Lang, Cesario Pancinha, Michelle C Williams, Steven Niederer, Shouvik Haldar, Mark O’Neill, Steven E Williams

**Affiliations:** Department of Cardiology, Guy’s and St Thomas’ Hospital & School of Biomedical Engineering and Imaging Sciences, King’s College London, Westminster Bridge Road, London SE1 7EH, UK; Institute for Neuroscience and Cardiovascular Research, 49 Little France Crescent, Edinburgh, EH16 4SB, University of Edinburgh, Edinburgh, UK; Heart Rhythm Management Centre, Universitair Ziekenhuis, Brussels, Belgium. Department of Electronics and Informatics (ETRO), Vrije Universiteit Brussel (VUB), Brussels, Belgium; Department of Cardiology, Guy’s and St Thomas’ Hospital & School of Biomedical Engineering and Imaging Sciences, King’s College London, Westminster Bridge Road, London SE1 7EH, UK; Department of Cardiology, Guy’s and St Thomas’ Hospital & School of Biomedical Engineering and Imaging Sciences, King’s College London, Westminster Bridge Road, London SE1 7EH, UK; Department of Cardiology, Guy’s and St Thomas’ Hospital & School of Biomedical Engineering and Imaging Sciences, King’s College London, Westminster Bridge Road, London SE1 7EH, UK; Institute for Neuroscience and Cardiovascular Research, 49 Little France Crescent, Edinburgh, EH16 4SB, University of Edinburgh, Edinburgh, UK; Institute for Neuroscience and Cardiovascular Research, 49 Little France Crescent, Edinburgh, EH16 4SB, University of Edinburgh, Edinburgh, UK; Department of Cardiology, St Thomas’ Hospital, Westminster Bridge Road, London SE1 7EH, UK, Guy’s and St Thomas’ NHS Foundation Trust, London, UK; Institute for Neuroscience and Cardiovascular Research, 49 Little France Crescent, Edinburgh, EH16 4SB, University of Edinburgh, Edinburgh, UK; Department of Cardiology, St Thomas’ Hospital, Westminster Bridge Road, London SE1 7EH, UK, Guy’s and St Thomas’ NHS Foundation Trust, London, UK; Department of Cardiology, Guy’s and St Thomas’ Hospital & School of Biomedical Engineering and Imaging Sciences, King’s College London, Westminster Bridge Road, London SE1 7EH, UK; Department of Cardiology, Guy’s and St Thomas’ Hospital & School of Biomedical Engineering and Imaging Sciences, King’s College London, Westminster Bridge Road, London SE1 7EH, UK; Department of Cardiology, St Thomas’ Hospital, Westminster Bridge Road, London SE1 7EH, UK, Guy’s and St Thomas’ NHS Foundation Trust, London, UK; Department of Cardiology, St Thomas’ Hospital, Westminster Bridge Road, London SE1 7EH, UK, Guy’s and St Thomas’ NHS Foundation Trust, London, UK; Department of Cardiology, St Thomas’ Hospital, Westminster Bridge Road, London SE1 7EH, UK, Guy’s and St Thomas’ NHS Foundation Trust, London, UK; Department of Cardiology, St Thomas’ Hospital, Westminster Bridge Road, London SE1 7EH, UK, Guy’s and St Thomas’ NHS Foundation Trust, London, UK; National Heart and Lung Institute, Dovehouse Street, London, SW3 6LY, Imperial College London, London W12 0NN, UK; Department of Cardiology, St. Vincent’s University Hospital Elm Park, Dublin, D04T6F4, Ireland; Heart Rhythm Centre, Harefield Hospital, Hill End Road, Harefield UB9 6JH, Royal Brompton & Harefield Hospitals, Guy’s and St Thomas’ NHS Foundation Trust, London, UK; Heart Rhythm Centre, Harefield Hospital, Hill End Road, Harefield UB9 6JH, Royal Brompton & Harefield Hospitals, Guy’s and St Thomas’ NHS Foundation Trust, London, UK; Department of Cardiology, Guy’s and St Thomas’ Hospital & School of Biomedical Engineering and Imaging Sciences, King’s College London, Westminster Bridge Road, London SE1 7EH, UK; Institute for Neuroscience and Cardiovascular Research, 49 Little France Crescent, Edinburgh, EH16 4SB, University of Edinburgh, Edinburgh, UK; Institute for Neuroscience and Cardiovascular Research, 49 Little France Crescent, Edinburgh, EH16 4SB, University of Edinburgh, Edinburgh, UK; Institute for Neuroscience and Cardiovascular Research, 49 Little France Crescent, Edinburgh, EH16 4SB, University of Edinburgh, Edinburgh, UK; Institute for Neuroscience and Cardiovascular Research, 49 Little France Crescent, Edinburgh, EH16 4SB, University of Edinburgh, Edinburgh, UK; National Heart and Lung Institute, Dovehouse Street, London, SW3 6LY, Imperial College London, London W12 0NN, UK; Heart Rhythm Centre, Harefield Hospital, Hill End Road, Harefield UB9 6JH, Royal Brompton & Harefield Hospitals, Guy’s and St Thomas’ NHS Foundation Trust, London, UK; National Heart and Lung Institute, Dovehouse Street, London, SW3 6LY, Imperial College London, London W12 0NN, UK; Department of Cardiology, Guy’s and St Thomas’ Hospital & School of Biomedical Engineering and Imaging Sciences, King’s College London, Westminster Bridge Road, London SE1 7EH, UK; Department of Cardiology, Guy’s and St Thomas’ Hospital & School of Biomedical Engineering and Imaging Sciences, King’s College London, Westminster Bridge Road, London SE1 7EH, UK; Institute for Neuroscience and Cardiovascular Research, 49 Little France Crescent, Edinburgh, EH16 4SB, University of Edinburgh, Edinburgh, UK

**Keywords:** Atrial fibrillation, Multimodal phenotyping, K-mean clustering, Catheter ablation

## Abstract

**Aims:**

The heterogeneity in response to catheter ablation amongst atrial fibrillation (AF) patients classified according to arrhythmia duration highlights the need for improved phenotyping. Multimodal assessment may better characterize the spectrum of pathophysiology present amongst patients classified as having paroxysmal or persistent AF. This study aimed to perform comprehensive multimodal pre-ablation phenotyping in patients undergoing AF ablation and evaluate associations with post-ablation arrhythmia recurrence, symptom burden, and bi-atrial substrate remodelling.

**Methods and results:**

In this multicentre prospective cohort study, 86 patients referred for first-time AF ablation (42% persistent AF) underwent pre-ablation phenotyping beyond usual care, incorporating patch-based ambulatory rhythm monitoring, atrial structural and functional MRI assessment, and formal symptom evaluation. Unsupervised *K*-means clustering was used to derive phenotypes. The primary outcome was 12-month arrhythmia recurrence; secondary analyses assessed symptom trajectories and bi-atrial substrate characteristics. Four AF phenotypes were identified. Arrhythmia recurrence occurred in 56.0%, 42.9%, 22.2%, and 19.0% of patients in Phenotypes 1–4, respectively (log-rank *P* = 0.032). Symptom trajectories differed across phenotypes, with the greatest improvements observed in Phenotypes 3 and 4. Exploratory electroanatomic mapping analyses demonstrated significant between-phenotype differences in bi-atrial bipolar voltage aligned with clinical trajectories. Importantly, phenotypes were not fully explained by conventional classification: Phenotype 2 experienced relatively high recurrence despite comprising predominantly paroxysmal AF patients whilst Phenotype 3 demonstrated favourable outcomes despite a high prevalence of persistent AF.

**Conclusion:**

Multimodal phenotyping identified distinct AF subgroups associated with differences in post-ablation recurrence, symptom trajectories, and electrophysiological substrate. This approach could improve pre-ablation risk stratification and shared decision-making.

**Registration:**

ClinicalTrials.gov Identifier: NCT05057507

## Graphical Abstract

**Graphical Abstract:**
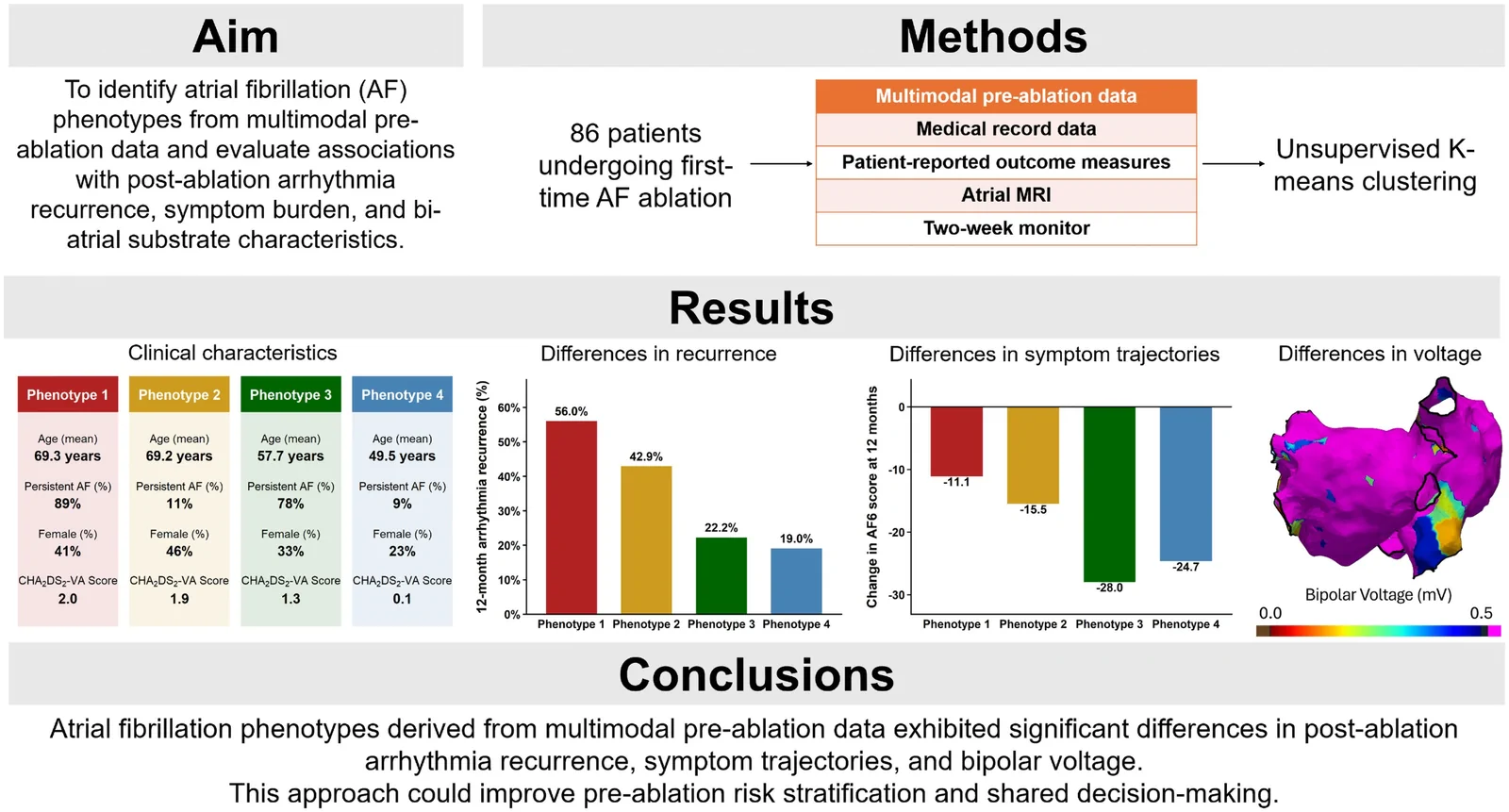

What's new

- To our knowledge, this is the first study to perform comprehensive, multimodal phenotyping incorporating clinical characteristics, patient-reported outcome measures, cardiac magnetic resonance imaging, and ambulatory rhythm monitoring in patients undergoing atrial fibrillation ablation.
- The identified phenotypes demonstrated distinct post-ablation clinical trajectories characterized by differences in post-ablation arrhythmia recurrence rates, symptom burden, and bi-atrial voltage.
- Multimodal pre-ablation phenotyping could confer improved risk stratification and patient selection for atrial fibrillation ablation procedures, provided the findings of the present study are confirmed in larger independent cohorts.

## Introduction

Atrial fibrillation is classified according to arrhythmia duration, with episodes lasting <7 days defined as paroxysmal and those lasting >7 days or requiring pharmacological or electrical cardioversion for termination classified as persistent.^1^ Catheter ablation is an established rhythm control strategy to alleviate symptoms and improve quality of life, although reported 1-year arrhythmia-free survival rates vary considerably, ranging from ∼45–60% in persistent atrial fibrillation to 70–90% in paroxysmal atrial fibrillation.^2–5^ The heterogeneity in response to ablation highlights the limitations of current classification methods which are based on arrhythmia duration, and emphasizes the requirement for novel approaches for disease phenotyping.

Prior to atrial fibrillation catheter ablation, large volumes of patient data can be acquired in both hospital and community settings, which may facilitate improved patient phenotyping. Clinical data relating to patient demographics and comorbidities are readily available from the medical record, whilst patient-reported outcome measures can be used to assess health status and arrhythmia-related quality of life.^6^ Advanced imaging modalities such as atrial magnetic resonance imaging can facilitate the accurate assessment of bi-atrial size and function,^7^ whilst ambulatory monitoring can be used to quantify pre-procedural atrial fibrillation burden. Collectively, the combination of these data modalities could provide a multidimensional quantification of symptomatology, arrhythmia burden, and atrial remodelling which could enable a more comprehensive and personalized approach towards atrial fibrillation phenotyping. However, such multimodality assessment is currently beyond usual care pathways and therefore requires evaluation in dedicated prospective studies.

We therefore hypothesized that a multimodal, data-driven approach to atrial fibrillation phenotyping could identify distinct patient phenotypes associated with post-ablation arrhythmia recurrence and symptom burden. The aim of this study was to integrate pre-procedural patient-reported quality of life measures, advanced cardiac imaging, ambulatory monitoring, and routinely collected clinical data to derive phenotypes and evaluate their association with arrhythmia recurrence and symptom burden following ablation. We further undertook detailed assessment of atrial electrophysiology during the ablation procedure to study the relationship between disease phenotypes and established invasive measures of atrial arrhythmogenic substrate.

## Methods

### Study design and sample

A multicentre, prospective, observational cohort study was conducted in patients referred for first-time atrial fibrillation catheter ablation between July 2022 and February 2025 across three study sites: St Thomas’ Hospital, Royal Infirmary of Edinburgh, and Harefield Hospital. Patients with paroxysmal atrial fibrillation or persistent atrial fibrillation of <24 months’ duration were eligible for inclusion, with full inclusion and exclusion criteria provided in Supplementary material online, *Table S1*. All patients provided written informed consent, and the study received a favourable opinion from the Health Research Authority and the North of Scotland Research Ethics Committee (21/NS/0080).

Medical record data, including patient demographics, comorbidities, and medication history, were documented. All patients underwent a detailed multimodal assessment additional to usual care comprising symptom questionnaires, 2-week patch-based ambulatory monitoring, and atrial imaging using cardiac magnetic resonance.

### Pre-procedural multimodal assessment

Quality of life was assessed prior to ablation and at 3 monthly intervals post-ablation for 1 year using the EQ-5D-5L tool together with the atrial fibrillation-specific Atrial Fibrillation Six (AF6) tool. The EQ-5D-5L is a multi-attribute health utility instrument which evaluates health across five dimensions (mobility, self-care, usual activities, pain/discomfort, and anxiety/depression) and includes a visual analogue scale (0–100).^8^ Responses to the five dimensions were converted to a single utility score using the EQ-5D-5L crosswalk to the UK EQ-5D-3L value set (1 = full health; 0 = death).^9^ The AF6 symptom scale is a disease-specific patient-reported outcome instrument which comprises six items (breathlessness at rest, breathlessness on exertion, limitations in daily life, discomfort, fatigue, and worry/anxiety attributable to atrial fibrillation), each scored on a numerical rating scale from 0 (no symptoms) to 10 (severe symptoms). Sum scores range from 0 to 60 with higher values reflecting greater atrial fibrillation symptom burden.^10^

Study participants underwent 14-day patch-based ambulatory electrocardiographic monitoring pre-ablation (Bardy CAM, Bardy Diagnostics, USA). Ambulatory recordings were used to quantify atrial fibrillation burden, which was defined as the percentage of monitored time spent in atrial fibrillation, expressed as a percentage, in accordance with contemporary consensus definitions.^11^

Pre-procedural atrial cardiac magnetic resonance imaging was performed using a standardized acquisition and post-processing protocol, as previously described.^12^ Circle cvi42 (v5.11.4, Cardiovascular Imaging, Calgary, Canada) was used to analyse cine images in two- and four-chamber views. Maximum and minimum atrial volumes were measured at end-systole and end-diastole using automatic contouring with manual adjustment when required. The monoplanar method was used to measure right atrial volumes from four-chamber images, whilst the biplanar method was used to measure left atrial volumes from two- and four-chamber views. Bi-atrial emptying fractions (Vmax-Vmin/Vmax) were subsequently calculated from right and left atrial volumetric measurements.^13^

### Unsupervised *K*-means clustering

Unsupervised clustering was performed to identify patient phenotypes using pre-ablation data only. Variables entered into clustering included age, body mass index, CHA_2_DS_2_-VA score, atrial fibrillation type at enrolment, pre-ablation atrial fibrillation burden on ambulatory monitoring, total pre-ablation AF6 symptom score, left atrial minimum volume, right atrial minimum volume, left atrial emptying fraction, and right atrial emptying fraction (*Figure 1*). Only variables available prior to catheter ablation and with near-complete availability across the cohort were included in the clustering model to maximize clinical applicability and minimize bias arising from incomplete data. Missing data across clustering variables were handled using median imputation. Variance inflation factor analysis was performed to assess for multicollinearity. Principal component analysis, an approach that transforms intercorrelated variables into a smaller number of principal components,^14^ was performed on standardized variables, and components explaining ≥70% of cumulative variance were retained. The number of subgroups was informed by elbow plot assessment^15^ of the total within-cluster sum of squares. Phenotype stability was assessed using 1000 bootstrap resamples and quantified using the Jaccard coefficient.^16^

**Figure 1:**
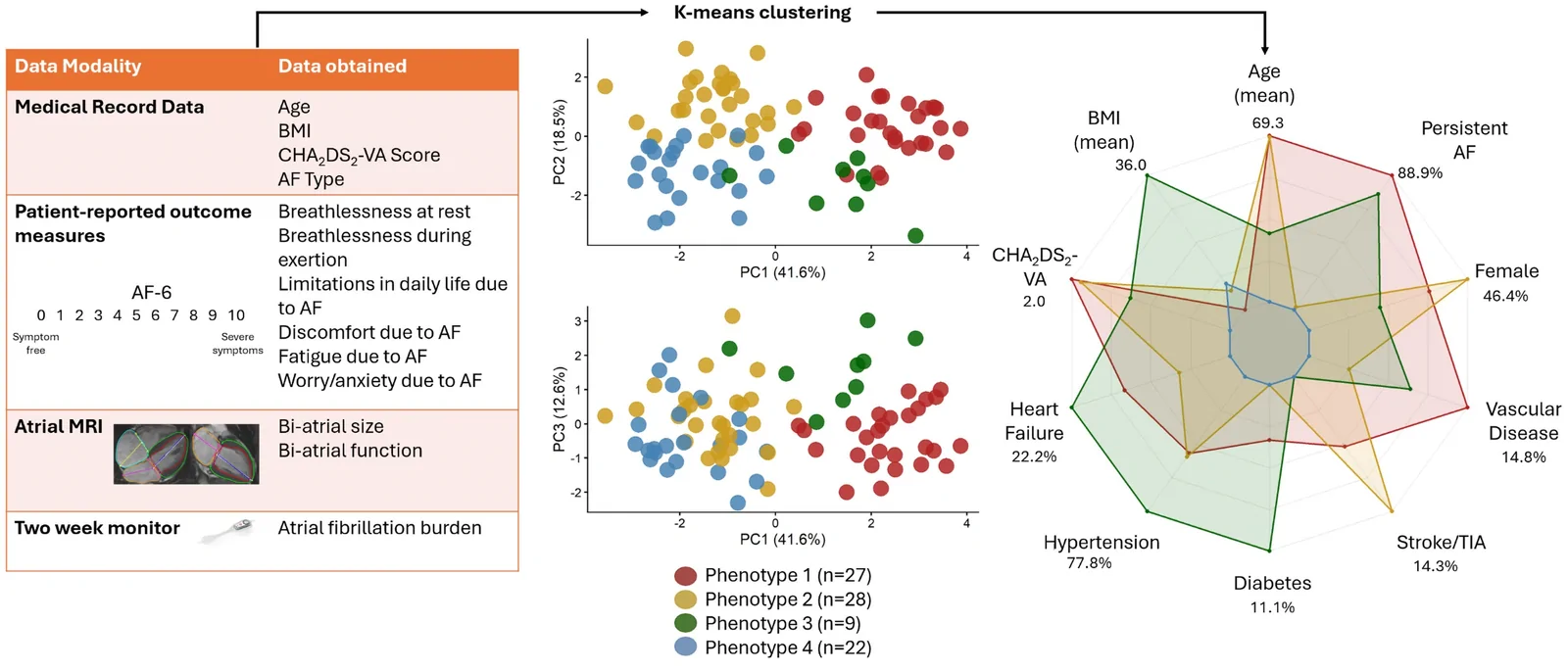
Multimodal phenotyping and cluster characterization of patients with atrial fibrillation. Clinical, imaging, and ambulatory monitoring data were integrated to derive patient phenotypes using an unsupervised clustering approach. (Left) Data modalities incorporated into the clustering model. (Middle) Principal component analysis of the integrated dataset demonstrating separation of patients into four phenotypes within principal component space. (Right) Radar plot illustrating the clinical characteristics of the four phenotypes.

### Atrial fibrillation catheter ablation

Atrial fibrillation catheter ablation was performed under either general anaesthesia or local anaesthesia with conscious sedation, in accordance with local institutional protocols. All antiarrhythmic drugs were discontinued at least five half-lives before the procedure. For patients who presented in atrial fibrillation, electrical cardioversion was performed to restore sinus rhythm prior to atrial mapping. Bi-atrial electroanatomic maps were acquired with PentaRay catheters using the CARTO 3 system (Biosense Webster) during proximal coronary sinus pacing at a cycle length of 500 ms. All patients were scheduled for pulmonary vein isolation only at enrolment; however, consistent with usual practice, operators could perform additional ablation if clinical circumstances changed either pre- or intra-procedurally. All patients who proceeded to ablation underwent radiofrequency catheter ablation.

### Bi-atrial substrate characterization

Magnetic resonance imaging-defined fibrosis burden (which is dependent upon adequate image quality^17^) and electroanatomic mapping parameters (which are only available intra-procedurally) were not included in the pre-ablation clustering model but were instead evaluated in exploratory analyses of substrate differences across phenotypes. Late gadolinium enhancement image quality assessment was performed by two expert observers prior to analysis using previously published visual quality scoring criteria.^18^ This method employs a five-point Likert scale where scores are assigned for myocardium nulling, sharpness quality, enhancement of fibrous tissue quality, and overall quality for atrial fibrosis assessment. Scans with an overall quality for atrial fibrosis assessment score of two or below were excluded from analysis (see Supplementary material online, *Figure S1*).

Quantitative atrial late gadolinium enhancement assessment was subsequently performed using CEMRGapp (Version 2.2, www.cemrg.com^19^ with aortic segmentation to exclude areas of high signal intensity arising from the aorta. Magnetic resonance imaging-defined fibrosis burden was quantified as the proportion of fibrotic tissue relative to the total left or right atrial surface area using the following thresholds: (i) Image Intensity Ratio (IIR) 0.97 × blood pool mean,^20^ (ii) IIR 1.2 × blood pool mean, and (iii) IIR 1.32 × blood pool mean.^21^

Electroanatomic maps were visually reviewed for chamber-wide anatomical coverage and point distribution, with mapping point counts and densities calculated. Electroanatomic maps demonstrating satisfactory chamber-wide anatomical coverage and point distribution without substantial unsampled atrial regions were retained for analysis.

OpenEP and EP Workbench, a desktop software platform which enables comprehensive quantitative analysis of electrophysiology data using the OpenEP algorithms,^22,23^ were utilized for quantitative mapping analysis. Prior to analysis, thoracic veins, valve annuli, and appendages were excluded and points with invalid activation times or located >1 mm from the atrial surface mesh were automatically removed. Points remaining were subsequently manually reviewed for inclusion using previously described criteria.^24^ This comprised review of the points to confirm atrial capture during coronary sinus pacing at a cycle length of 500 ms. Points which contained ventricular far-field activity or noise were excluded. Signal morphology was then evaluated to distinguish near-field from far-field electrograms. Near-field signals were defined as high-frequency electrograms demonstrating at least two sharp peaks, as previously described.^24^ The width of the signal was visually assessed to confirm high-frequency characteristics, and electrogram morphology was evaluated for temporal and spatial consistency with adjacent mapping points. Signals lacking sharp peaks, demonstrating instability, or inconsistent with neighbouring electrograms were classified as far-field or uncertain and were excluded from further analysis.

Quantitative mapping analysis was performed to assess bi-atrial mean bipolar voltage, low-voltage area percentage, mean conduction velocity, and wave collision percentage.^22,23^ Further details regarding the methodology used to calculate these parameters are provided in the Supplementary material.

### Study outcomes

The primary outcome was 12-month atrial arrhythmia recurrence following ablation. Recurrence was defined as documented atrial fibrillation lasting >30 s, or patient-reported symptoms corroborated by electrocardiographic documentation, occurring after an 8-week blanking period.^25^ Changes in EQ-5D-5L and AF6 scores at 3, 6, 9, and 12 months were assessed as pre-specified secondary outcomes. Additional sensitivity analyses of both arrhythmia recurrence and patient-reported outcomes were conducted in patients undergoing pulmonary vein isolation ± cavotricuspid isthmus ablation only. Exploratory analyses were performed to assess the association between phenotype membership and measures of atrial substrate, including bi-atrial electroanatomic mapping parameters and magnetic resonance imaging-defined fibrosis burden.

### Statistical analysis

Baseline characteristics were summarized by phenotype descriptively. Continuous variables are presented as means or medians, as appropriate, and categorical variables as counts and percentages. Differences in the distribution of individual ablation lesion sets across phenotypes were assessed using Fisher’s exact test. Freedom from atrial arrhythmia recurrence was assessed using Kaplan–Meier analysis and compared across phenotypes using the log-rank test. Associations between phenotype membership and time-to-recurrence were assessed using Cox proportional hazards regression. The proportional hazards assumption was assessed using Schoenfeld residual testing.

For patient-reported outcomes, EQ-5D-5L utility, EQ-Visual Analogue Scale, and AF6 scores and their changes from baseline at 3, 6, 9, and 12 months were compared across phenotypes using the Kruskal–Wallis test. Electroanatomic mapping parameters were compared across phenotypes separately for the left and right atria using the Kruskal–Wallis test. Statistical analyses were performed in R (R Foundation for Statistical Computing, Vienna, Austria), and a two-sided *P* < 0.05 was considered statistically significant.

## Results

### Study population

Ninety-one patients were recruited to the study with five patients excluded prior to analysis (one without cardiac magnetic resonance imaging and four who were removed from the waiting list prior to ablation). The final cohort therefore comprised 86 patients. Baseline characteristics of the study population are presented in *Table 1*. Missing data across variables included in the clustering model were minimal (0.1%), attributable to a single unreturned 2-week patch-based monitor.

**Table 1:** Baseline patient characteristics stratified by patient phenotype

Variable	Overall (*n* = 86)	Phenotype 1 (*n* = 27)	Phenotype 2 (*n* = 28)	Phenotype 3 (*n* = 9)	Phenotype 4 (*n* = 22)	*P*-value
Enrolment age (years)	63.0 ± 11.6	69.3 ± 6.9	69.2 ± 6.5	57.7 ± 5.4	49.5 ± 10.9	<0.001
Female sex	32 (37.2%)	11 (40.7%)	13 (46.4%)	3 (33.3%)	5 (22.7%)	0.362
BMI (kg/m^2^)	27.7 ± 5.3	25.5 ± 2.8	27.0 ± 4.3	36.0 ± 4.8	27.6 ± 6.0	<0.001
Persistent AF	36 (41.9%)	24 (88.9%)	3 (10.7%)	7 (77.8%)	2 (9.1%)	<0.001
Congestive cardiac failure	8 (9.3%)	4 (14.8%)	2 (7.1%)	2 (22.2%)	0 (0%)	0.123
Hypertension	36 (41.9%)	13 (48.1%)	14 (50%)	7 (77.8%)	2 (9.1%)	<0.001
Stroke/TIA/thromboembolism	6 (7.0%)	2 (7.4%)	4 (14.3%)	0 (0%)	0 (0%)	0.296
Vascular disease	8 (9.3%)	4 (14.8%)	2 (7.1%)	1 (11.1%)	1 (4.5%)	0.617
Diabetes	2 (2.3%)	1 (3.7%)	0 (0%)	1 (11.1%)	0 (0%)	0.195
CHA_2_DS_2_-VA	1.4 ± 1.2	2.0 ± 1.1	1.9 ± 1.2	1.3 ± 0.9	0.1 ± 0.4	<0.001
Duration of persistent AF (months)	14.6 ± 5.6	14.5 ± 5.0	17.2 ± 6.0	11.9 ± 8.1	16.3 ± 3.8	0.472
Number of prior cardioversions	0.4 ± 0.6	0.7 ± 0.6	0.1 ± 0.3	0.6 ± 0.5	0.3 ± 0.6	<0.001
Pre-procedural AF burden (%)	43.2 ± 45.9	93.8 ± 18.1	12.4 ± 22.3	69.9 ± 45.9	8.1 ± 22.0	<0.001
Medications at the time of study enrolment						
Beta blocker	60 (69.8%)	23 (85.2%)	17 (60.7%)	6 (66.7%)	14 (63.6%)	0.191
Digoxin	7 (8.1%)	4 (14.8%)	1 (3.6%)	2 (22.2%)	0 (0%)	0.047
Calcium channel blocker	5 (5.8%)	0 (0%)	3 (10.7%)	1 (11.1%)	1 (4.5%)	0.26
NOAC	72 (83.7%)	25 (92.6%)	28 (100%)	9 (100%)	10 (45.5%)	<0.001
Vitamin K antagonist	1 (1.2%)	1 (3.7%)	0 (0%)	0 (0%)	0 (0%)	1
Flecainide	20 (23.3%)	1 (3.7%)	8 (28.6%)	0 (0%)	11 (50%)	<0.001
Sotalol	2 (2.3%)	0 (0%)	1 (3.6%)	0 (0%)	1 (4.5%)	0.793
Propafenone	1 (1.2%)	0 (0%)	0 (0%)	0 (0%)	1 (4.5%)	0.359
Dronedarone	4 (4.7%)	1 (3.7%)	2 (7.1%)	1 (11.1%)	0 (0%)	0.579

BMI, body mass index; TIA, transient ischaemic attack; NOAC, non-vitamin K oral anticoagulant.

### Phenotype creation

All included variables had variance inflation factors < 5 indicating no evidence of problematic collinearity (see Supplementary material online, *Table S2*).^26^ Principal component analysis of pre-ablation variables yielded three principal components explaining ≥70% of cumulative variance, which were retained for *K*-means clustering. The first component primarily reflected atrial remodelling, with positive loadings for atrial fibrillation burden and bi-atrial volumes and negative loadings for atrial emptying fraction. The second component was driven by clinical risk factors, whilst the third component was associated with body mass index and symptom burden. Full component loadings are provided in Supplementary material online, *Table S3*.

The optimal number of phenotypes was determined using the elbow method based on total within-cluster sum of squares. The largest curvature in the elbow curve occurred at *k* = 4, supported by the maximal second difference in reduction of within-cluster sum of squared distances (26.7), and this value was therefore selected (see Supplementary material online, *Figure S2*). Bootstrapped Jaccard coefficients are reported in Supplementary material online, *Table S4*. Mean bootstrap Jaccard coefficients exceeded 0.70 for all phenotypes, indicating at least moderate cluster stability.^27^

### Phenotype characteristics

Baseline characteristics for the four phenotypes are depicted in *Table 1* and *Figure 1*. Phenotype 1 (*n* = 27) was characterized by older age (69.3 ± 6.9 years), a high prevalence of persistent atrial fibrillation (88.9%) (*Figure 2*), and a greater overall comorbidity burden, reflected by higher CHA_2_DS_2_-VA scores (2.0 ± 1.1). This phenotype also demonstrated the largest bi-atrial volumes and the lowest atrial emptying fractions, consistent with more advanced structural remodelling (*Table 2*).

**Figure 2:**
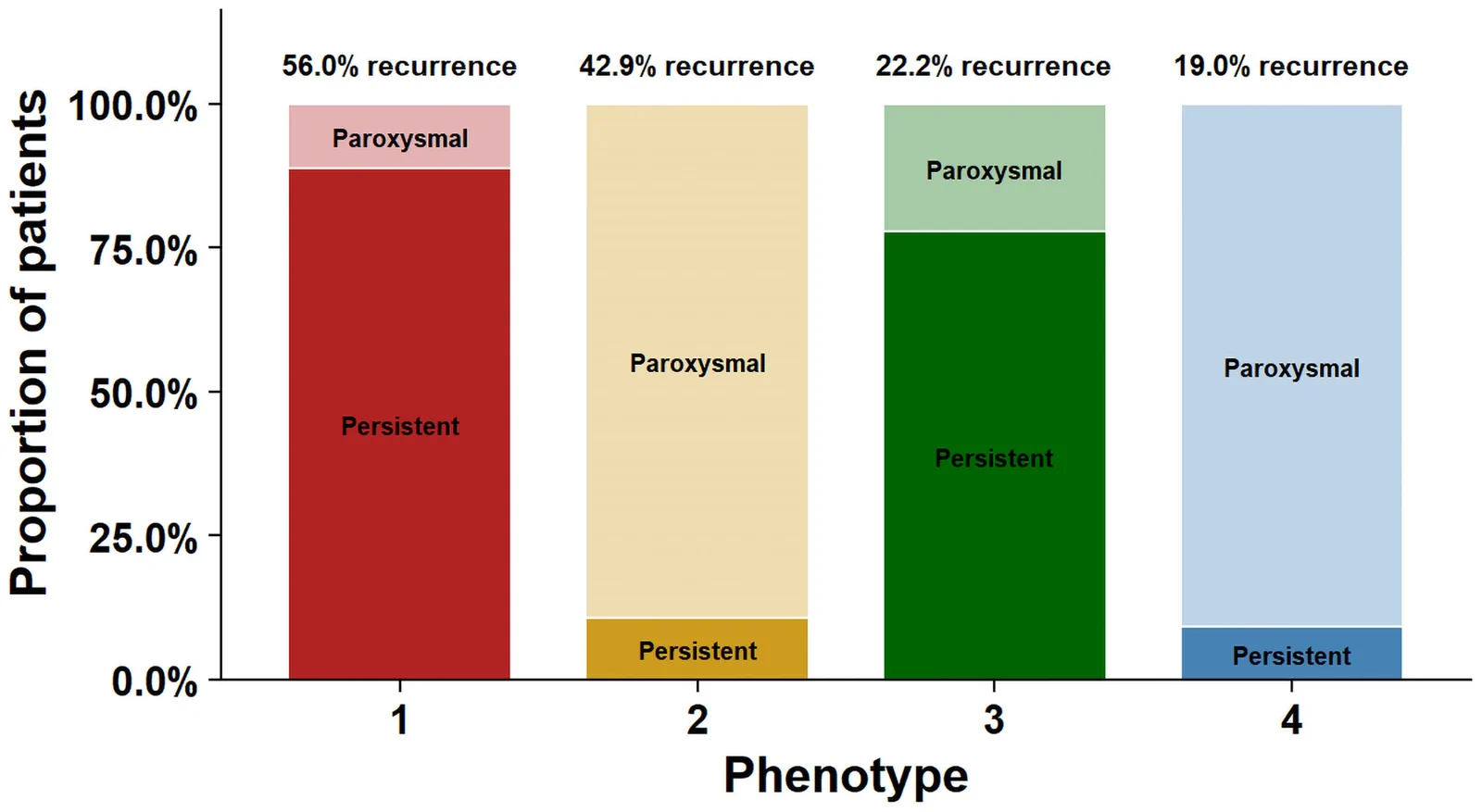
Stacked bar charts demonstrating the proportions of patients with paroxysmal and persistent atrial fibrillation within each phenotype. Colours denote phenotype membership, with lighter shades representing paroxysmal atrial fibrillation and darker shades representing persistent atrial fibrillation. The percentage of patients experiencing arrhythmia recurrence at 12 months is shown above each bar.

**Table 2:** Magnetic resonance imaging parameters describing chamber size and function stratified by patient phenotype

Variable	Overall (*n* = 86)	Phenotype 1 (*n* = 27)	Phenotype 2 (*n* = 28)	Phenotype 3 (*n* = 9)	Phenotype 4 (*n* = 22)	*P*-value
LA area (cm^2^)	28.02 ± 5.69	30.81 ± 5.69	26.54 ± 4.96	31.22 ± 5.14	25.32 ± 4.98	0.001
RA area (cm^2^)	26.16 ± 5.66	29.31 ± 5.27	24.68 ± 5.39	26.56 ± 5.39	24.18 ± 5.22	0.005
LA Vmin (mL)	63.66 ± 35.24	93.98 ± 35.53	45.38 ± 17.69	89.79 ± 28.66	39.01 ± 13.87	<0.001
LA Vmin (mL/m^2^)	32.24 ± 18.20	49.04 ± 18.60	23.50 ± 9.55	40.58 ± 14.65	19.33 ± 6.65	<0.001
LA Vmax (mL)	98.39 ± 35.01	117.84 ± 41.69	85.55 ± 26.14	116.22 ± 28.55	83.56 ± 23.39	<0.001
LA Vmax indexed (mL/m^2^)	49.76 ± 18.41	61.58 ± 22.13	44.16 ± 14.12	52.31 ± 14.87	41.32 ± 11.21	<0.001
LA emptying fraction (%)	39.78 ± 18.55	21.91 ± 9.05	49.21 ± 13.78	24.73 ± 10.39	55.86 ± 10.39	<0.001
RA Vmin (mL)	66.21 ± 29.17	88.31 ± 29.80	47.59 ± 18.96	78.16 ± 23.44	57.88 ± 21.18	<0.001
RA Vmin (mL/m^2^)	33.28 ± 14.52	45.80 ± 14.36	24.67 ± 10.37	34.57 ± 9.28	28.36 ± 10.03	<0.001
RA Vmax (mL)	107.97 ± 35.21	124.83 ± 35.80	98.58 ± 31.45	113.45 ± 34.87	96.97 ± 32.75	0.017
RA Vmax indexed (mL/m^2^)	54.43 ± 18.17	65.04 ± 18.15	50.88 ± 17.00	50.52 ± 15.70	47.52 ± 15.62	0.004
RA emptying fraction (%)	41.70 ± 13.58	31.05 ± 7.33	53.91 ± 9.32	33.57 ± 14.73	42.56 ± 10.59	<0.001
LV ejection fraction (%)	55.65 ± 9.51	49.96 ± 9.04	60.46 ± 8.00	53.50 ± 10.18	57.10 ± 8.19	<0.001
RV ejection fraction (%)	54.30 ± 11.15	49.31 ± 7.33	57.93 ± 5.46	50.12 ± 6.64	57.24 ± 17.89	<0.001

LA, left atrium; RA, right atrium; Vmin, minimum volume; Vmax, maximum volume.

Phenotype 2 (*n* = 28) comprised the highest proportion of female patients (46.4%) but a low prevalence of persistent atrial fibrillation (10.7%), with hypertension present in 50% of patients. These patients exhibited smaller atrial volumes and preserved atrial function.

Phenotype 3 (*n* = 9) included patients with the highest body mass index (36.0 ± 4.8 kg/m^2^) and a high prevalence of persistent atrial fibrillation (77.8%). Whilst this group demonstrated a greater prevalence of hypertension (77.8%) and heart failure (22.2%) compared with other phenotypes, they also exhibited lower average CHA_2_DS_2_-VA scores compared with Phenotypes 1 and 2 indicating a lower overall comorbidity burden. Although this group displayed impaired bi-atrial function, indexed atrial volumes were less dilated than Phenotype 1.

Phenotype 4 (*n* = 22) consisted of younger patients (49.5 ± 10.9 years) with low rates of persistent atrial fibrillation (9.1%) and the lowest comorbidity burden with no recorded heart failure, diabetes, or cerebrovascular disease. This phenotype demonstrated smaller atrial volumes and higher atrial emptying fractions, consistent with a less remodelled substrate.

### Arrhythmia recurrence

Pulmonary vein isolation ± cavotricuspid isthmus ablation was performed in 89.4% of patients who underwent atrial fibrillation catheter ablation (see Supplementary material online, *Table S5*). No significant differences in the distribution of ablation lesion sets were observed between phenotypes (all *P* > 0.05). Two patients who underwent pulmonary vein isolation alone were lost to follow-up during the 12-month follow-up period.

Arrhythmia recurrence occurred in 56.0%, 42.9%, 22.2%, and 19.0% of patients in Phenotypes 1–4, respectively (*Figure 2*). The proportional hazards assumption was not violated. Kaplan–Meier analysis demonstrated significant differences in freedom from atrial arrhythmia recurrence across phenotypes (log-rank *P* = 0.032) (*Figure 3A*). In Cox regression analysis using Phenotype 1 as the reference group, Phenotype 4 demonstrated a significantly lower hazard of arrhythmia recurrence (hazard ratio 0.23, 95% confidence interval 0.08–0.72; *P* = 0.011) (see Supplementary material online, *Table S6*). Similar findings were observed in a sensitivity analysis restricted to patients undergoing pulmonary vein isolation (±cavotricuspid isthmus ablation) only (log-rank *P* = 0.021) (*Figure 3B*).

**Figure 3:**
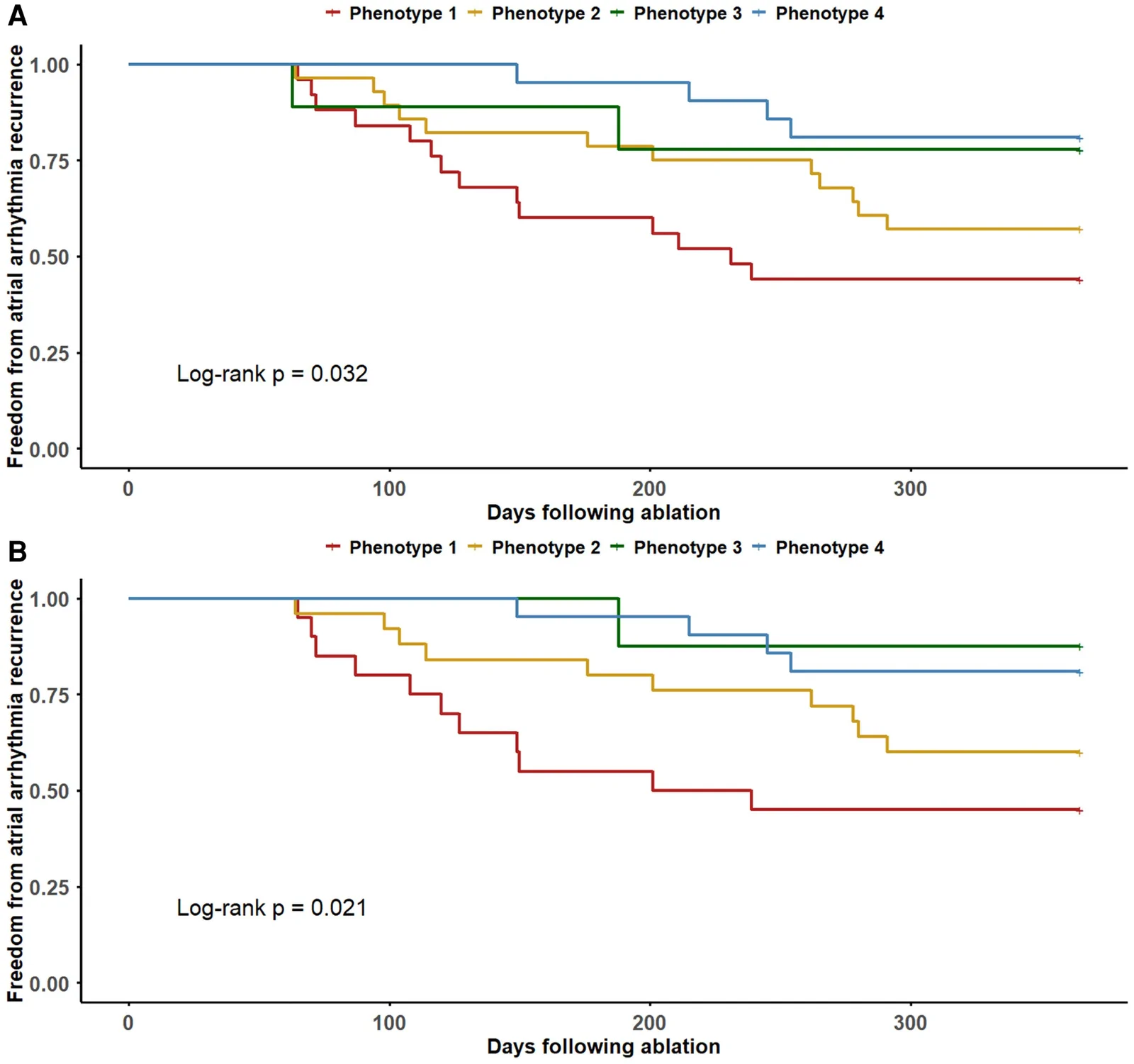
Kaplan–Meier analysis stratified by patient phenotype in (*A*) all patients who underwent atrial fibrillation catheter ablation and (*B*) patients who underwent pulmonary vein isolation ± cavotricuspid isthmus ablation.

### Symptom questionnaires

Patient-reported outcomes following ablation differed across phenotypes over the 12-month follow-up period (see Supplementary material online, *Table S7*). Baseline AF6 scores differed significantly between phenotypes (*P* < 0.001), with higher symptom burdens in Phenotypes 3 and 4. Although improvements in atrial fibrillation-related symptoms were observed across all phenotypes following ablation (*Figure 4A*), the magnitude of improvement differed significantly between phenotypes at all time points (all *P* < 0.001). Absolute AF6 scores were no longer significantly different between phenotypes at 3, 6, 9, or 12 months (all *P* > 0.05), consistent with greater absolute improvement in the more symptomatic phenotypes. At 12 months, the greatest reductions in AF6 score were observed in Phenotypes 3 and 4 (−28.0 ± 13.3 and −24.7 ± 10.0, respectively), compared with more modest improvements in Phenotypes 1 and 2 (−11.1 ± 10.8 and −15.5 ± 10.8, respectively). Similar findings were observed in a sensitivity analysis restricted to patients undergoing pulmonary vein isolation (±cavotricuspid isthmus ablation) only (see Supplementary material online, *Figure S3*).

**Figure 4:**
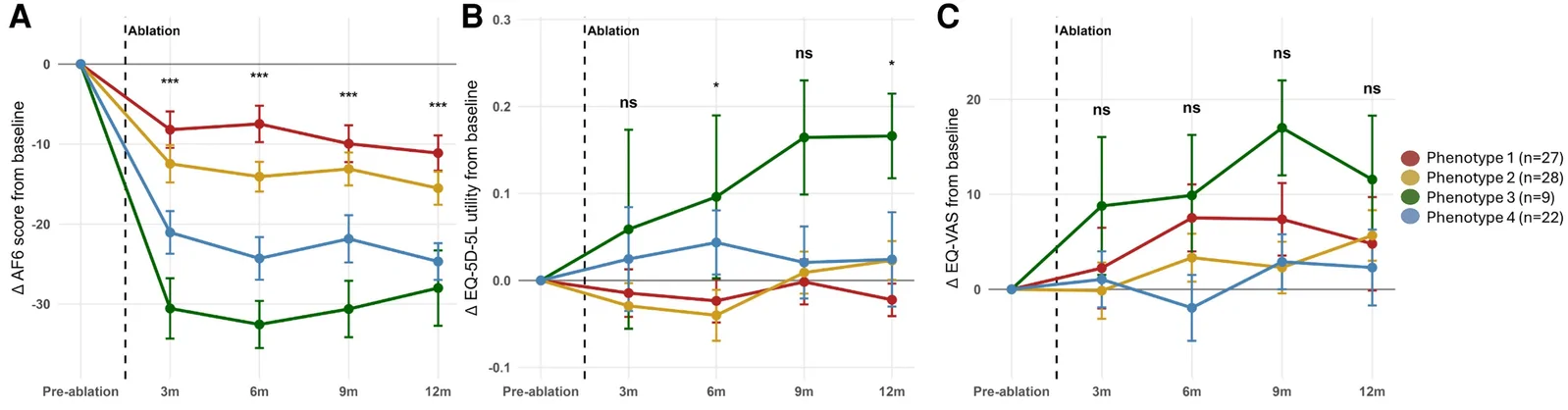
Changes in patient-reported outcomes following atrial fibrillation ablation across phenotypes. Temporal changes in patient-reported outcome measures following catheter ablation are shown for each phenotype. The dashed vertical line indicates the timing of ablation. Values represent mean change from baseline with error bars indicating standard error. (*A*) Change in AF6 symptom score. (*B*) Change in EQ-5D-5L utility index. (*C*) Change in EQ-VAS score from baseline over 12 months. Differences between phenotypes were assessed using the Kruskal–Wallis test with significance depicted as follows: ns (*P* > 0.05), * (*P* < 0.05), ** (*P* ≤ 0.01), and *** (*P* ≤ 0.001).

Improvements in EQ-5D-5L utility varied across phenotypes, with the greatest increases observed in Phenotype 3 (*Figure 4B*). Baseline EQ-5D-5L utility differed between phenotypes (*P* = 0.009), but absolute values were similar at follow-up, with between-phenotype differences in change from baseline reaching statistical significance at 6 and 12 months (*P* < 0.05). In contrast, changes in EQ-Visual Analogue Scale scores did not differ significantly between phenotypes at any follow-up time point (all *P* > 0.05) (*Figure 4C*).

### Bi-atrial substrate characteristics

Bi-atrial substrate characteristics are presented in *Table 3* and *Figure 5*. Electroanatomic maps were quantitatively analysed in 65/86 patients (76%) for the left atrium (mean number of left atrial mapping points: 2702 ± 1141; mean point density: 25.9 ± 11.7 points/cm^2^) and in 63/86 patients (73%) for the right atrium (mean number of right atrial mapping points: 2055 ± 842; mean point density: 18.2 ± 8.6 points/cm^2^). Across phenotypes, significant differences were observed in both left and right atrial mean bipolar voltage and low-voltage area percentage, with Phenotype 1 exhibiting both the lowest mean bipolar voltages and highest proportion of low-voltage areas. In contrast, there were no significant differences in mean conduction velocity or wave collision percentage between phenotypes.

**Figure 5:**
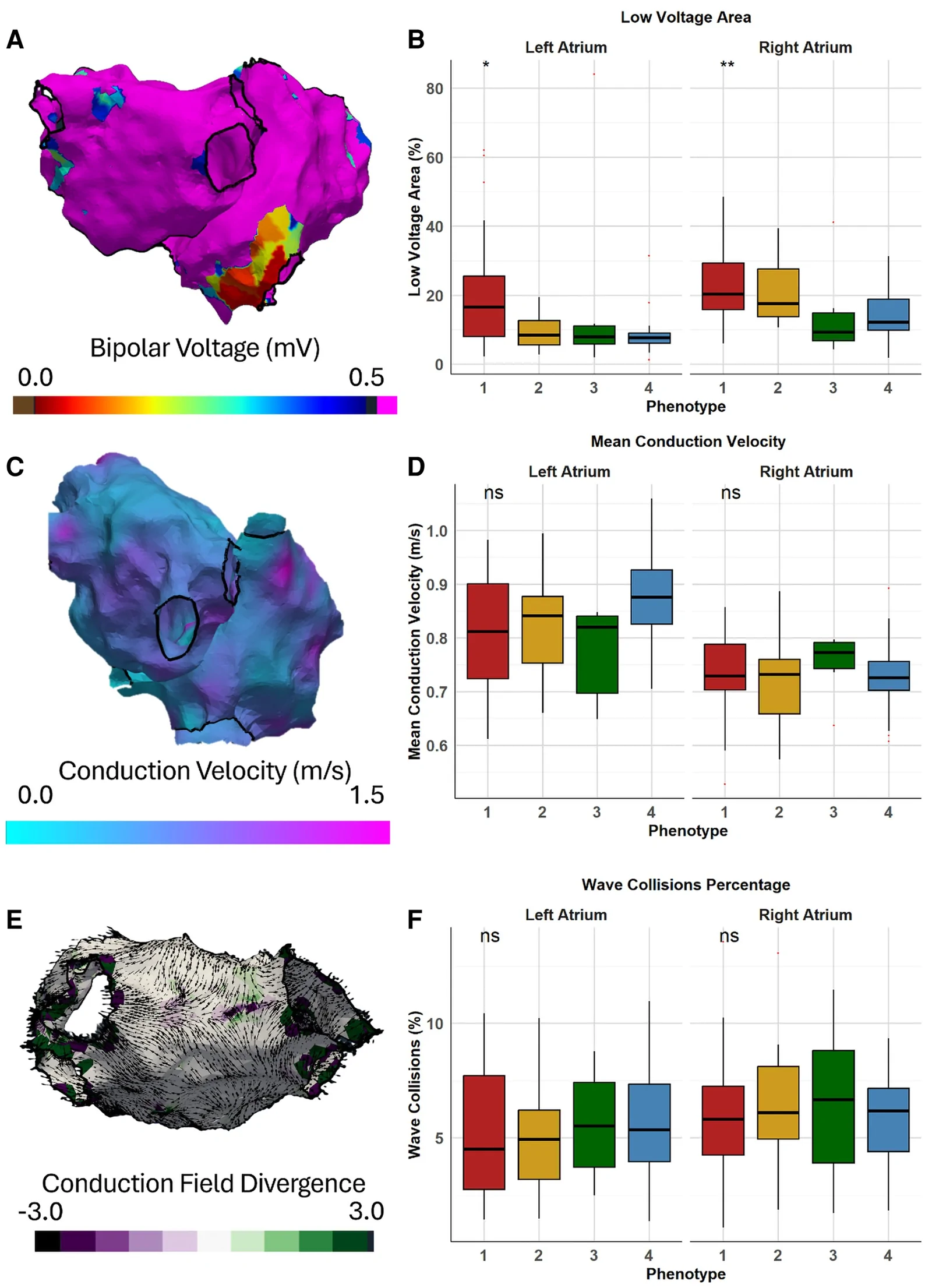
Electroanatomic mapping parameters across atrial fibrillation phenotypes. (*A*) Bi-atrial bipolar voltage map. (*B*) Low-voltage area percentage across phenotypes for the left and right atria. (*C*) Bi-atrial conduction velocity map. (*D*) Mean conduction velocity (m/s) across phenotypes. (*E*) Left atrial conduction field divergence map. Regions with a divergence value of <−1.5 were classified as wave collision points. (*F*) Wave collision percentage across phenotypes for the left and right atria. Differences between phenotypes were assessed using the Kruskal–Wallis test with significance depicted as follows: ns (*P* > 0.05), * (*P* < 0.05), ** (*P* ≤ 0.01), and *** (*P* ≤ 0.001).

**Table 3:** Bi-atrial substrate characteristics stratified by patient phenotype

Variable	Phenotype 1 (*n* = 27)	Phenotype 2 (*n* = 28)	Phenotype 3 (*n* = 9)	Phenotype 4 (*n* = 22)	*P*-value
Electroanatomic mapping					
Left atrium					
Electroanatomic map available	21 (78%)	20 (71%)	6 (67%)	18 (82%)	
Low-voltage area (%)	21.66 ± 18.32	9.25 ± 4.28	19.91 ± 31.56	8.89 ± 6.64	0.046
Mean bipolar voltage (mV)	1.54 ± 0.56	2.17 ± 0.48	1.93 ± 1.15	2.75 ± 0.76	<0.001
Mean conduction velocity (m/s)	0.816 ± 0.109	0.819 ± 0.094	0.773 ± 0.094	0.877 ± 0.084	0.126
Wave collision (%)	5.14 ± 2.85	4.87 ± 2.35	5.57 ± 2.49	5.68 ± 2.67	0.787
Right atrium					
Electroanatomic map available	21 (78%)	19 (68%)	6 (67%)	17 (77%)	
Low-voltage area (%)	23.18 ± 10.23	21.5 ± 8.9	14.46 ± 13.67	14.06 ± 7.25	0.006
Mean bipolar voltage (mV)	1.98 ± 0.59	2.01 ± 0.50	2.46 ± 0.82	2.46 ± 0.64	0.046
Mean conduction velocity (m/s)	0.687 ± 0.073	0.671 ± 0.083	0.698 ± 0.071	0.683 ± 0.073	0.752
Wave collision (%)	5.89 ± 3.08	6.25 ± 2.67	6.5 ± 3.67	5.74 ± 2.23	0.908
Late gadolinium enhancement magnetic resonance imaging					
Left atrium					
Fibrosis assessment quality > 2	23 (89%)	23 (82%)	5 (56%)	19 (86%)	
LGE IIR 0.97	38.50 ± 9.04	40.16 ± 8.54	36.50 ± 9.07	41.59 ± 7.55	0.478
LGE IIR 1.20	6.82 ± 6.30	5.51 ± 4.82	6.35 ± 5.66	5.74 ± 2.80	0.533
LGE IIR 1.32	2.45 ± 3.79	1.40 ± 2.16	2.19 ± 2.96	1.64 ± 1.45	0.203
Right atrium					
Fibrosis assessment quality > 2	20 (77%)	22 (79%)	5 (56%)	17 (77%)	
LGE IIR 0.97	38.65 ± 9.12	36.77 ± 8.65	38.00 ± 8.15	40.75 ± 6.16	0.481
LGE IIR 1.20	10.48 ± 7.67	8.95 ± 5.34	8.82 ± 5.54	11.04 ± 9.46	0.384
LGE IIR 1.32	4.33 ± 5.49	2.65 ± 2.76	3.18 ± 3.15	5.42 ± 6.59	0.353

Percentages for fibrosis assessment quality are calculated amongst patients with available LGE imaging.

LGE, late gadolinium enhancement; IIR, image intensity ratio.

Respiratory-navigated late gadolinium enhancement imaging was performed in 85 of 86 patients, with one study not completed due to prolonged acquisition time for ECG-gated three-dimensional late gadolinium enhancement imaging. Following image quality assessment, left atrial fibrosis was analysed in 70/85 (82%) patients and right atrial fibrosis in 64/85 (75%) patients. There were no significant differences in magnetic resonance imaging-defined fibrosis burden across phenotypes at any of the image intensity ratio thresholds tested (*Table 3*).

## Discussion

In this multicentre, prospective cohort study, we performed comprehensive multimodal pre-ablation phenotyping to identify four distinct subgroups in patients undergoing first-time atrial fibrillation ablation procedures. The phenotypes demonstrated significant differences in (i) arrhythmia recurrence following ablation, (ii) post-ablation symptom improvement, and (iii) bi-atrial bipolar voltage on electroanatomic mapping, providing evidence to suggest that the derived phenotypes captured variation in both procedural outcomes and the underlying atrial fibrillation substrate.

Whilst prior clustering studies using registry and electronic health record datasets have also identified subgroups organized by age and comorbidity burden,^28^ they have typically not incorporated detailed multimodal, pre-procedural phenotyping. By integrating symptom burden, ambulatory atrial fibrillation burden, and atrial magnetic resonance imaging, our approach identified four distinct phenotypes amongst patients previously classified with either paroxysmal atrial fibrillation or persistent atrial fibrillation lasting <24 months.

Phenotype 1 represented an older, comorbid population with a high prevalence of persistent atrial fibrillation and the lowest bi-atrial voltages, consistent with more advanced atrial remodelling, higher arrhythmia recurrence following ablation, and more modest improvements in symptom burden. In contrast, Phenotype 4 comprised younger patients with minimal comorbidity burden and comparatively preserved atrial substrate characteristics, who demonstrated both favourable procedural outcomes and substantial symptom improvement. These favourable outcomes were supported by magnetic resonance imaging findings, with Phenotype 4 demonstrating smaller bi-atrial volumes and higher atrial emptying fractions. These findings would suggest that this phenotype displays features consistent with moderate atrial cardiomyopathy according to consensus guidelines, which include the manifestation of atrial fibrillation as a defining characteristic.^29^ This phenotype may represent a younger, low-comorbidity form of atrial fibrillation, broadly analogous to the historically described ‘lone atrial fibrillation’ subgroup, and this finding is consistent with prior clustering studies that have also identified younger, low-comorbidity atrial fibrillation phenotypes.^30–33^

An important observation was that Phenotype 2 exhibited relatively high recurrence rates and modest post-ablation symptom improvement in comparison to Phenotypes 3 and 4 despite a high prevalence of paroxysmal atrial fibrillation. This group also exhibited relative preservation of bi-atrial size and function, findings that are consistent with moderate atrial cardiomyopathy.^29^ The reasons for the comparatively poorer outcomes in Phenotype 2 remain unclear but may reflect the contribution of comorbidities as this phenotype displayed a higher average CHA_2_DS_2_-VA score than Phenotypes 3 and 4.

Interestingly, Phenotype 3 demonstrated lower recurrence rates and substantial symptom improvement despite a high prevalence of persistent atrial fibrillation and elevated body mass index. Phenotype 3 was characterized by a lower average age and CHA_2_DS_2_-VA score compared with Phenotypes 1 and 2, which may partly explain the more favourable outcomes observed. Compared with Phenotype 1, Phenotype 3 also demonstrated a shorter duration of persistent atrial fibrillation and smaller bi-atrial size, suggesting a less advanced degree of atrial remodelling. Although both Phenotypes 1 and 3 had a higher number of prior cardioversions compared with Phenotypes 2 and 4, consistent with a greater prevalence of persistent atrial fibrillation, pre-procedural atrial fibrillation burden was lower in Phenotype 3 than in Phenotype 1, which may also reflect differences in responsiveness to cardioversion. These findings may suggest that Phenotype 3 represents a subgroup with established but potentially less advanced atrial remodelling and that there may be a spectrum of pathophysiology amongst patients uniformly classified as having persistent atrial fibrillation.

Differences in the clinical trajectories of Phenotype 1 and Phenotype 3, which both comprised relatively higher proportions of patients with persistent atrial fibrillation, could also relate to differences in the underlying aetiology of atrial cardiomyopathy. Although both groups demonstrated atrial systolic failure (markedly reduced left atrial emptying fraction ≤ 35%) which would be consistent with severe atrial cardiomyopathy,^29^ it is plausible to speculate that ageing may have contributed more substantially to Phenotype 1 and obesity-related metabolic remodelling to Phenotype 3. Ageing and obesity are thought to promote atrial cardiomyopathy through overlapping but potentially distinct pathways, with obesity additionally associated with metabolic dysfunction, epicardial adipose tissue expansion, and inflammation.^29^ These processes may result in differing patterns of atrial remodelling which could contribute to the distinct clinical characteristics and outcomes observed between these phenotypes. Further work is therefore justified to distinguish the biological processes underpinning atrial cardiomyopathy in distinct clinical contexts.

Principal component analysis provided insight into the mechanistic basis of these phenotypes. The first component primarily reflected atrial remodelling, with positive loadings for atrial fibrillation burden and bi-atrial volumes and negative loadings for atrial emptying fraction, whilst the second component was driven by clinical risk factors, including age and CHA_2_DS_2_-VA score. These findings highlight how multimodal data can be combined to capture the heterogeneity of atrial fibrillation through the combination of markers of atrial remodelling with clinical risk factors.

An important observation from our study was the discordance between bi-atrial voltage and late gadolinium enhancement, with significant differences in voltage observed despite the absence of corresponding differences in late gadolinium enhancement. Differences in their ability to characterize the extent and spatial distribution of pathological substrate have previously been reported.^34,35^ Our findings may therefore reflect differences in the sensitivity of the two techniques to different patterns of fibrosis. In cardiac tissue, interstitial fibrosis is characterized by collagen deposition within the interstitium, occurring either between muscle bundles (perimysial fibrosis) or between individual cardiomyocytes (endomysial fibrosis).^36^ Experimental evidence suggests that endomysial fibrosis, rather than overall fibrosis burden, is the strongest determinant of atrial fibrillation complexity,^37^ indicating that the microstructural pattern of fibrosis may be more important than its total extent. Voltage mapping may therefore be detecting differences in endomysial fibrosis that are not reflected by differences in the overall extent of late gadolinium enhancement, as previously proposed by Sim *et al.*^38^

Additionally, small areas of patchy or interstitial arrhythmogenic fibrosis may fall below the spatial resolution of contemporary late gadolinium enhancement imaging,^39^ rendering them undetectable on magnetic resonance imaging despite producing measurable alterations in local bipolar voltage. Consequently, electroanatomic mapping may identify functionally relevant microstructural remodelling that is not captured by current magnetic resonance imaging.

Importantly, however, the observed discordance should not necessarily be attributed to differences in fibrosis alone as both modalities can be influenced by other pathological processes. For example, increased late gadolinium enhancement uptake can also reflect amyloid deposition and inflammation,^40^ whilst bipolar voltage has been associated with histopathological changes, including increased intercellular space, myofibrillar loss, and reduced nuclear density.^41^ Furthermore, the observed differences in bipolar voltage may also reflect differences in the clinical characteristics of the identified phenotypes. A prior systematic review demonstrated that low-voltage areas are associated with multiple clinical, imaging, and biomarker variables, highlighting the contribution of numerous factors to their development.^42^ Differences in patient demographics, comorbidity profiles, and imaging parameters amongst the phenotypes may therefore have additionally contributed to the observed differences in bipolar voltage.

Whilst further studies are required, the methodology presented in this study may support a more phenotype-guided approach to rhythm control intervention, with potential relevance to patient selection prior to catheter ablation. Current guidelines highlight the heterogeneity of atrial fibrillation and emphasize the need for personalized risk stratification and patient selection for rhythm control strategies.^1^ In the present study, multimodal pre-ablation data were combined to identify phenotypes associated with differing procedural outcomes. If our findings are validated in larger independent cohorts, the approach presented may eventually contribute to improved risk stratification and support more informed shared decision-making between clinicians and patients prior to catheter ablation.

The findings of the present study support a widely held clinical view that conventional risk markers may be more relevant when interpreted in the context of the broader clinical phenotype. Moreover, our approach provides an evidence-based, practical strategy to achieve this in the clinical setting. Moving beyond simple, single-modality treatment-eligibility checkpoints is likely to be necessary to support appropriate prescription of therapies and to avoid limiting access to therapies based on single parameters alone, such as atrial size or BMI.

### Limitations

Several limitations should be acknowledged. First, the achievable sample size was limited, and these findings require confirmation in larger cohorts. Second, although the study was conducted across multiple centres, the identified phenotypes were derived within a single study population and were not validated in a second cohort. The findings of the present study therefore require external validation. Third, electroanatomic mapping and late gadolinium enhancement data, which were not included in the clustering model, were not available in all participants due to stringent acquisition and quality-control criteria. Analyses of bi-atrial substrate characteristics should therefore be considered exploratory. Finally, the study cohort comprised patients undergoing radiofrequency ablation, and the generalizability of these findings to other ablation modalities, including cryoballoon or pulsed field ablation, remains to be established.

### Conclusion

In this multicentre, prospective cohort study, comprehensive, multimodal pre-ablation phenotyping incorporating clinical characteristics, patient-reported outcome measures, cardiac magnetic resonance imaging, and ambulatory rhythm monitoring identified four distinct atrial fibrillation subgroups with differing arrhythmia recurrence rates and symptom trajectories following catheter ablation. Although the identified phenotypes require validation in larger independent cohorts, these findings highlight the potential of multimodal, data-driven phenotyping to capture the heterogeneity of atrial fibrillation and support improved risk stratification and shared decision-making.

## Supplementary Material

oeag162_Supplementary_Data

## Data Availability

The dataset studied and analysed during the present study is not publicly available due to the presence of potentially sensitive personal information. However, de-identified data may be made available from the corresponding author on reasonable request, subject to approval by a relevant institutional review board or ethics committee.
