# Supplementary material for: Atrial fibrillation phenotypes derived from multimodal data exhibit distinct electrophysiological substrate characteristics, symptom trajectories, and ablation outcomes
Source: Eur Heart J Open. 2026 Sep 28;6(6):oeag162. doi: 10.1093/ehjopen/oeag162 (PMC13728345; doi:10.1093/ehjopen/oeag162)
Supplement: Supplementary file 1 [file oeag162_supplementary_data.docx]

# Supplementary Material

## Supplementary Methods - Atrial fibrillation catheter ablation procedures

Catheter ablation procedures were allocated an additional 45 minutes to permit comprehensive bi-atrial electroanatomic mapping. To minimise repeated cardioversions, no more than four research direct current cardioversions were permitted per participant. If this threshold was reached, research measurements were ceased and the procedure continued according to standard clinical practice.

Conduction velocity was derived from local activation times interpolated across the atrial surface using a radial basis function approach, as previously described.[1] Wave collision points were identified by assessing the divergence of the conduction velocity vector field, with negative divergence values indicating areas of wavefront collision.[2] Regions with a divergence value of < −1.5 were classified as wave collision points and were excluded from subsequent conduction velocity analyses.[3] Voltage maps were created in EP Workbench from the raw electrogram signals using nearest neighbour interpolation.[4] A bipolar voltage threshold of 0.5mV was used to define tissue as low voltage and the proportion of low voltage areas were calculated by indexing the low voltage area to the total mapped left/right atrial surface area.

## Supplementary Tables/Figures

Supplementary Table S1. Inclusion/Exclusion Criteria

| Inclusion Criteria | Exclusion Criteria |
| --- | --- |
| (1) Symptomatic paroxysmal atrial fibrillation or persistent atrial fibrillation diagnosed within 24 months preceding the consent date. (2) Plan at the time of enrolment to proceed to atrial fibrillation ablation following clinical consultation consisting of pulmonary vein isolation (3) Able and willing to comply with study follow-up requirements (4) Age 18-80 years (5) Able and willing to provide written informed consent | - Any clinical contra-indication to ablation - Any disease limiting life expectancy to < 1year - Contra-indication to MRI including renal dysfunction (eGFR<30ml/min) - Potential participant currently pregnant or breast feeding - Prior ablation, cardiac surgery or presence of any prosthetic valves - Myocardial infarction or percutaneous coronary intervention within 3 months preceding consent date - Hypertrophic cardiomyopathy or other inherited cardiac condition - Presence / likely implant of any pacemaker, cardiac resynchronization therapy device or implantable defibrillator within 1 year - Enrolment in an existing interventional atrial fibrillation study which alters treatment or follow-up. - Use of amiodarone for any indication within 6 months prior to the planned ablation procedure - Use of any anti-arrhythmic drug either within five half-lives of the ablation procedure or for an arrhythmia indication other than atrial fibrillation - Cavotricuspid isthmus dependent right atrial flutter documented prior to study enrolment with planned cavotricuspid isthmus ablation to be performed at the time of atrial fibrillation ablation - Unable to understand verbal or written explanations given in English. |

Supplementary Table S2. Variance inflation factor (VIF) analysis.

| Variable | VIF |
| --- | --- |
| Age | 2.53 |
| Body mass index | 1.24 |
| CHA_2_DS_2_-VA | 1.99 |
| Atrial fibrillation type | 2.74 |
| Pre-procedural atrial fibrillation burden | 4.90 |
| AF6 Total Score | 1.27 |
| Left atrial volume | 2.93 |
| Right atrial volume | 2.06 |
| Left atrial emptying fraction | 4.75 |
| Right atrial emptying fraction | 2.45 |

Supplementary Table S3. Loadings for the three principal components (PCs) retained for clustering. Principal components were derived from scaled pre-ablation clinical, imaging, ambulatory monitoring, and patient-reported variables. Components explaining ≥70% cumulative variance were retained.

| Variable | PC1 | PC2 | PC3 |
| --- | --- | --- | --- |
| Age | 0.178 | 0.611 | 0.140 |
| Body mass index | -0.004 | -0.269 | 0.660 |
| CHA_2_DS_2_-VA | 0.195 | 0.489 | 0.307 |
| Atrial fibrillation type | -0.397 | 0.030 | 0.004 |
| Pre-procedural atrial fibrillation burden | 0.441 | -0.044 | 0.013 |
| AF6 Total Score | -0.106 | -0.367 | 0.517 |
| Left atrial volume | 0.407 | -0.056 | 0.158 |
| Right atrial volume | 0.319 | -0.246 | -0.314 |
| Left atrial emptying fraction | -0.434 | -0.002 | -0.154 |
| Right atrial emptying fraction | -0.333 | 0.337 | 0.188 |

Supplementary Table S4. Bootstrapped Jaccard Coefficients.

| Phenotype | Number of bootstraps | Median Jaccard | Interquartile Range | Mean Jaccard |
| --- | --- | --- | --- | --- |
| 1 | 1000 | 0.813 | 0.722-0.917 | 0.804±0.137 |
| 2 | 1000 | 0.813 | 0.722-0.900 | 0.808±0.125 |
| 3 | 1000 | 0.938 | 0.842-1.000 | 0.902±0.113 |
| 4 | 1000 | 0.857 | 0.545-1.000 | 0.734±0.312 |

Supplementary Table S5. Atrial fibrillation ablation lesion sets (n = 85). One patient did not undergo ablation due to the development of a pericardial effusion following bi-atrial electroanatomic mapping. PVI = pulmonary vein isolation; CTI = cavotricuspid isthmus.

| Ablation lesion set | Total | Phenotype 1 | Phenotype 2 | Phenotype 3 | Phenotype 4 | p value |
| --- | --- | --- | --- | --- | --- | --- |
| PVI | 65 (76.5%) | 20 (74.1%) | 20 (71.4%) | 8 (88.9%) | 17 (77.3%) | 0.823 |
| PVI + CTI | 11 (12.9%) | 1 (3.7%) | 5 (17.9%) | 0 (0.0%) | 5 (22.7%) | 0.123 |
| PVI + CTI + Posterior wall isolation + Mitral isthmus ablation | 1 (1.2%) | 0 (0.0%) | 1 (3.6%) | 0 (0.0%) | 0 (0.0%) | 1.000 |
| PVI + CTI + Posterior wall isolation | 1 (1.2%) | 1 (3.7%) | 0 (0.0%) | 0 (0.0%) | 0 (0.0%) | 0.674 |
| PVI + Posterior wall isolation | 5 (5.9%) | 2 (7.4%) | 2 (7.1%) | 1 (11.1%) | 0 (0.0%) | 0.517 |
| PVI + Posterior wall isolation + Vein of Marshall ethanol infusion | 1 (1.2%) | 1 (3.7%) | 0 (0.0%) | 0 (0.0%) | 0 (0.0%) | 0.674 |
| Posterior wall isolation only | 1 (1.2%) | 1 (3.7%) | 0 (0.0%) | 0 (0.0%) | 0 (0.0%) | 0.674 |

Supplementary Table S6. Cox proportional hazards analysis of atrial arrhythmia recurrence by phenotype using Phenotype 1 as the reference group. PVI = Pulmonary Vein Isolation; CTI = Cavotricuspid isthmus.

| Ablation Lesion Set Cohort | Comparison | Hazard Ratio | 95% Confidence Interval | P value |
| --- | --- | --- | --- | --- |
| All lesion sets | Phenotype 2 vs Phenotype 1 | 0.61 | 0.28-1.33 | 0.215 |
| All lesion sets | Phenotype 3 vs Phenotype 1 | 0.31 | 0.07-1.35 | 0.117 |
| All lesion sets | Phenotype 4 vs Phenotype 1 | 0.23 | 0.08-0.72 | 0.011 |
| PVI ± CTI | Phenotype 2 vs Phenotype 1 | 0.55 | 0.23-1.31 | 0.179 |
| PVI ± CTI | Phenotype 3 vs Phenotype 1 | 0.15 | 0.02-1.17 | 0.070 |
| PVI ± CTI | Phenotype 4 vs Phenotype 1 | 0.23 | 0.07-0.73 | 0.012 |

Supplementary Table S7. Longitudinal patient-reported outcome measures stratified by phenotype. Values are presented as mean (SD). Between-phenotype comparisons were performed using the Kruskal–Wallis test.

| Measure | Row | Phenotype 1 mean (SD) | Phenotype 1 n | Phenotype 2 mean (SD) | Phenotype 2 n | Phenotype 3 mean (SD) | Phenotype 3 n | Phenotype 4 mean (SD) | Phenotype 4 n | Between-phenotype p-value |
| --- | --- | --- | --- | --- | --- | --- | --- | --- | --- | --- |
| EQ-5D-5L Utility | Baseline | 0.884 (0.144) | 27 | 0.86 (0.12) | 28 | 0.672 (0.153) | 9 | 0.819 (0.255) | 22 | 0.009 |
| EQ-5D-5L Utility | 3 months | 0.887 (0.097) | 25 | 0.831 (0.189) | 28 | 0.731 (0.34) | 9 | 0.84 (0.289) | 20 | 0.373 |
| EQ-5D-5L Utility | 6 months | 0.878 (0.106) | 25 | 0.823 (0.164) | 27 | 0.768 (0.281) | 9 | 0.857 (0.245) | 20 | 0.42 |
| EQ-5D-5L Utility | 9 months | 0.895 (0.128) | 24 | 0.868 (0.14) | 27 | 0.837 (0.14) | 8 | 0.853 (0.268) | 19 | 0.574 |
| EQ-5D-5L Utility | 12 months | 0.882 (0.145) | 24 | 0.882 (0.133) | 27 | 0.838 (0.143) | 9 | 0.876 (0.224) | 18 | 0.56 |
| EQ-5D-5L Utility | Change at 3 months | -0.015 (0.136) | 25 | -0.029 (0.138) | 28 | 0.059 (0.344) | 9 | 0.025 (0.267) | 20 | 0.072 |
| EQ-5D-5L Utility | Change at 6 months | -0.024 (0.125) | 25 | -0.04 (0.152) | 27 | 0.096 (0.281) | 9 | 0.044 (0.165) | 20 | 0.025 |
| EQ-5D-5L Utility | Change at 9 months | -0.002 (0.127) | 24 | 0.009 (0.125) | 27 | 0.164 (0.186) | 8 | 0.021 (0.181) | 19 | 0.085 |
| EQ-5D-5L Utility | Change at 12 months | -0.022 (0.091) | 24 | 0.023 (0.115) | 27 | 0.166 (0.146) | 9 | 0.024 (0.23) | 18 | 0.034 |
| EQ-VAS | Baseline | 67 (17.7) | 27 | 72.5 (10.3) | 28 | 57.3 (12.6) | 9 | 75.7 (19.1) | 21 | 0.018 |
| EQ-VAS | 3 months | 70.4 (19.3) | 25 | 72.4 (10.5) | 28 | 66.1 (15.2) | 9 | 77.1 (13.7) | 21 | 0.203 |
| EQ-VAS | 6 months | 75.7 (13.4) | 25 | 75.8 (11.5) | 27 | 67.2 (16.2) | 9 | 74.3 (15.5) | 20 | 0.516 |
| EQ-VAS | 9 months | 77.2 (13.9) | 24 | 75.7 (11.9) | 27 | 74.3 (14.3) | 7 | 78.3 (17.6) | 20 | 0.632 |
| EQ-VAS | 12 months | 72.5 (20.7) | 24 | 79 (10.7) | 27 | 68.9 (13.2) | 9 | 79.9 (16.4) | 18 | 0.126 |
| EQ-VAS | Change at 3 months | 2.2 (21.2) | 25 | -0.1 (15.7) | 28 | 8.8 (21.8) | 9 | 1 (13.2) | 20 | 0.81 |
| EQ-VAS | Change at 6 months | 7.5 (17.6) | 25 | 3.3 (13.1) | 27 | 9.9 (19.1) | 9 | -1.9 (15.1) | 19 | 0.141 |
| EQ-VAS | Change at 9 months | 7.4 (18.7) | 24 | 2.3 (14.1) | 27 | 17 (13.2) | 7 | 2.9 (12.6) | 19 | 0.109 |
| EQ-VAS | Change at 12 months | 4.8 (24.1) | 24 | 5.7 (13.8) | 27 | 11.6 (20.2) | 9 | 2.3 (16.5) | 17 | 0.291 |
| AF6 Total Score | Baseline | 17.9 (9.1) | 27 | 21.4 (10.8) | 28 | 40.6 (7.3) | 9 | 30.1 (12.2) | 22 | <0.001 |
| AF6 Total Score | 3 months | 10 (9.5) | 25 | 8.9 (8.4) | 28 | 10 (13.8) | 9 | 8.4 (10.5) | 21 | 0.906 |
| AF6 Total Score | 6 months | 10.8 (8.8) | 25 | 8 (7.7) | 27 | 8 (6.9) | 9 | 7 (10) | 20 | 0.268 |
| AF6 Total Score | 9 months | 8.3 (9.6) | 24 | 8.1 (9.9) | 27 | 10.8 (10.9) | 8 | 8.8 (12.8) | 20 | 0.926 |
| AF6 Total Score | 12 months | 6.6 (6.7) | 24 | 5.7 (5.4) | 27 | 12.5 (15) | 8 | 8.1 (11) | 19 | 0.983 |
| AF6 Total Score | Change at 3 months | -8.2 (11.4) | 25 | -12.5 (12.3) | 28 | -30.6 (11.3) | 9 | -21 (12.2) | 21 | <0.001 |
| AF6 Total Score | Change at 6 months | -7.5 (11.4) | 25 | -14.1 (9.7) | 27 | -32.6 (8.8) | 9 | -24.3 (11.9) | 20 | <0.001 |
| AF6 Total Score | Change at 9 months | -10 (11.3) | 24 | -13.1 (10.7) | 27 | -30.6 (10) | 8 | -21.9 (13.2) | 20 | <0.001 |
| AF6 Total Score | Change at 12 months | -11.1 (10.8) | 24 | -15.5 (10.8) | 27 | -28 (13.3) | 8 | -24.7 (10) | 19 | <0.001 |

Supplementary Figure S1. Distribution of cardiac magnetic resonance image quality scores across the study cohort based on previously published image quality assessment criteria.[5] Bar charts show the frequency of scores (1–5) for each image quality domain, including myocardium nulling, image sharpness, enhancement of fibrous tissue, and suitability for left and right atrial fibrosis assessment.

Alternative text: Bar charts showing the distribution of cardiac magnetic resonance image quality scores across the study cohort for each image quality domain. Separate charts display the frequency of scores from 1 to 5 for myocardium nulling, image sharpness, enhancement of fibrous tissue, and suitability for left and right atrial fibrosis assessment.

Supplementary Figure S2. Elbow plot to determine optimal cluster number.

Alternative Text: Line graph showing the relationship between the number of clusters and the within-cluster sum of squares. The curve demonstrates an elbow, indicating the selected optimal number of clusters for the unsupervised clustering analysis.

Supplementary Figure S3. Changes in patient-reported outcomes following atrial fibrillation ablation across phenotypes in a sensitivity analysis restricted to patients undergoing pulmonary vein isolation ± cavotricuspid isthmus ablation. Temporal changes in patient-reported outcome measures following catheter ablation are shown for each phenotype. The dashed vertical line indicates the timing of ablation. Values represent mean change from baseline with error bars indicating standard error. (A) Change in AF6 symptom score from baseline over time; (B) Change in EQ-5D-5L utility index from baseline over time; (C) Change in EQ-VAS score from baseline over time. Differences between phenotypes were assessed using the Kruskal-Wallis test with significance depicted as follows: ns (p>0.05), * (p<0.05), ** (p≤0.01), and *** (p≤0.001).

Alternative text: Three line graphs showing changes in patient-reported outcomes from baseline following atrial fibrillation catheter ablation across the phenotypes in a sensitivity analysis restricted to patients undergoing pulmonary vein isolation ± cavotricuspid isthmus ablation. Panel A shows AF6 symptom score, Panel B shows EQ-5D-5L utility index, and Panel C shows EQ-VAS score. Mean changes from baseline are displayed with standard error bars, and a dashed vertical line indicates the timing of ablation. Statistical significance between phenotypes at each time point is indicated by asterisks.

# References

1.	Gharaviri A, Vigneswaran V, Vickneson K *et al.* Performance of atrial conduction velocity algorithms with error-prone clinical measurements for the identification of atrial fibrosis. *Comput Biol Med* 2025;**191**. https://doi.org/10.1016/j.compbiomed.2025.110119.

2.	Frontera A, Pagani S, Limite LR *et al.* Slow Conduction Corridors and Pivot Sites Characterize the Electrical Remodeling in Atrial Fibrillation. *JACC Clin Electrophysiol* 2022;**8**(5):561–77. https://doi.org/10.1016/J.JACEP.2022.01.019.

3.	Klis M, Gharaviri ; A, Vigneswaran ; V *et al.* Heterogenous atrial conduction in patients with hypertrophic cardiomyopathy and atrial fibrillation. *EP Europace* 2024;**26**(Supplement_1). https://doi.org/10.1093/EUROPACE/EUAE102.006.

4.	NearestNDInterpolator — SciPy v1.16.2 Manual. n.d. https://docs.scipy.org/doc/scipy/reference/generated/scipy.interpolate.NearestNDInterpolator.html#scipy.interpolate.NearestNDInterpolator (19 Dec. 2025, date last accessed).

5.	Orkild B, Arefeen Sultan KM, Kholmovski E *et al.* Image quality assessment and automation in late gadolinium-enhanced MRI of the left atrium in atrial fibrillation patients. *Journal of Interventional Cardiac Electrophysiology* 2025;**68**(3):667–79. https://doi.org/10.1007/S10840-024-01971-Z,.
